# Should each university department have only one postgraduate program?

**DOI:** 10.5935/0004-2749.2025-0019

**Published:** 2025-11-04

**Authors:** Newton Kara-Junior, Marcony R. Santhiago, Luiz Ubirajara Sennes, Mário Luiz Ribeiro-Monteiro

**Affiliations:** 1 Departamento de Oftalmologia e Otorrinolaringologia, Faculdade de Medicina, Universidade de São Paulo, São Paulo, SP, Brazil

Universities are complex institutions. To operate efficiently, there must be dynamic interaction between the various sectors and units. Within this structure, departments represent the smallest organizational units responsible for academic, scientific, and administrative functions. They coordinate the allocation of faculty and oversee teaching and research activities within specific knowledge domains^(1)^.

In medical education, the relevance and efficacy of departmental organizational structures are increasingly coming into question. This is partially due to the increasing need for greater integration across disciplines. As a result, some of the newer medical schools have replaced departments with “study sectors,” which are considered a more flexible and interdisciplinary approach to grouping faculty. Similarly, many of the older, more traditional institutions are considering administrative reforms that will reduce or eliminate rigid departmental boundaries.

However, in the context of research and postgraduate education, the reasoning differs. Individual disciplines within a department often pursue academic autonomy and scientific independence; yet, consolidating postgraduate academic efforts into a single program can bring substantial benefits. Uniting faculty, students, and infrastructure fosters a synergy that strengthens research activity. This collaborative approach is highly valued by CAPES, the agency responsible for evaluating academic postgraduate programs in Brazil. CAPES assessments serve as a strategic guide for postgraduate program development. They have shown that several key advantages emerge when departments concentrate their efforts into a single postgraduate program. These include:

- Enhanced interdisciplinary collaboration: A unified program promotes interaction between faculty from diverse but related fields. This fosters a more integrated and productive academic environment that benefits both teaching and research. Ultimately, this leads to higher quality output by both students and faculty.
- Improved research productivity: Concentrating resources, infrastructure, and intellectual effort increases the impact and visibility of scientific output. A stronger academic profile, in turn, attracts research funding, international collaboration, and top-tier students.
- Facilitation of international partnerships: When a faculty member has ties to an international institution, it becomes easier for colleagues within the same program to establish similar connections, thereby amplifying the department’s global reach.

From a strategic standpoint, we argue that there are measurable advantages to combining the efforts of related disciplines. These outweigh the convenience of maintaining multiple, fragmented postgraduate programs. A consolidated, high-quality postgraduate program can significantly influence the allocation of institutional resources, enhance the department’s standing within the university, and support the recruitment and development of faculty.

However, not all of the benefits of consolidation are immediately measurable. One of the main challenges in maintaining a highly rated program is achieving consistently high faculty productivity. Accreditation standards often require homogeneous faculty performance. This can lead to the exclusion of less productive members and increase program vulnerability; for example, when key faculty retire. In contrast, programs with a broader academic focus allow a larger, more balanced faculty. This facilitates generational renewal and the inclusion of promising young researchers without compromising stringent performance criteria.

However, while the model of a single postgraduate program per department offers clear advantages, it may not suit every institutional context. Maintaining separate programs may better address specific academic needs in departments with more diverse areas of expertise. Conversely, smaller departments may achieve better outcomes by participating in larger, interdisciplinary programs that transcend departmental boundaries.

In conclusion, the structure of academic postgraduate education should be guided by the specific characteristics, goals, and capacities of each department and institution. Nonetheless, in most cases, the consolidation of postgraduate efforts into a single, well-structured program can enhance academic quality, operational efficiency, and institutional visibility.

## References

[1] Favero MLA. Da cátedra universitária ao departamento:
subsídios para discussão May 2023. Gestus - Caderno de Administração e Gestão
Pública.

